# Recurrent colorectal liver metastasis patients could benefit from repeat hepatic resection

**DOI:** 10.1186/s12893-021-01323-y

**Published:** 2021-08-16

**Authors:** Wei Liu, Jia-Ming Liu, Kun Wang, Hong-Wei Wang, Bao-Cai Xing

**Affiliations:** Hepatopancreatobiliary Surgery Department I, Key Laboratory of Carcinogenesis and Translational Research, Ministry of Education, Peking University School of Oncology, Beijing Cancer Hospital and Institute, No. 52, Fu-Cheng-Lu Street, Beijing, 100142 People’s Republic of China

**Keywords:** CRLM, Recurrence, Repeat, Resection, RFA

## Abstract

**Background:**

Local treatment remains the best option for recurrent colorectal liver metastasis (CRLM). The current study aimed to investigate predictive factors of survival outcomes and select candidates for local treatment for CRLM at first recurrence.

**Methods:**

Data were collected retrospectively from CRLM patients who underwent hepatic resection and developed first recurrence between 2000 and 2019 at our institution. A nomogram predicting overall survival was established based on a multivariable Cox model of clinicopathologic factors. The predictive accuracy and discriminative ability of the nomogram were determined by the concordance index and calibration curve.

**Results:**

Among 867 patients who underwent curative hepatic resection, 549 patients developed recurrence. Three hundred patients were evaluated and had resectable and liver-limited disease. Among them, repeat liver resection and percutaneous radiofrequency ablation were performed in 88 and 85 patients, respectively. The other 127 patients received only systemic chemotherapy. Multivariable analysis identified primary lymph node positivity, tumor size > 3 cm, early recurrence, RAS gene mutation and no local treatment as independent risk factors for survival outcomes. Integrating these five variables, the nomogram presented a good concordance index of 0.707. Compared with patients who received only systemic chemotherapy, radical local treatment did not significantly improve survival outcomes (median OS: 21 vs. 15 months, p = 0.126) in the high-risk group (total score ≥ 13).

**Conclusion:**

Radical local treatment improved the survival of recurrent CRLM patients. The proposed model facilitates personalized assessments of prognosis for patients who develop first recurrence in the liver.

**Supplementary Information:**

The online version contains supplementary material available at 10.1186/s12893-021-01323-y.

## Key summary

More than half of CRLM patients develop recurrence after hepatic resection. The feasibility and safety of repeat local treatment have been tested. The present study created a nomogram to select recurrent CRLM patients who would benefit from repeat local treatment. This tool will help to design a personalized treatment regimen for recurrent CRLM.

## Background

The liver is the most common site of colorectal cancer metastasis, and approximately 50% of patients develop liver metastasis at some point during their disease course [1]. Hepatic resection is a potentially curative treatment for colorectal cancer liver metastasis (CRLM) patients with 5-year survival rates of 30–50% [2]. Unfortunately, more than half of CRLM patients recur after hepatic resection, and the majority of such recurrences occur within 2 years [3].

Recent advances in surgical approaches have dramatically changed the treatment strategy for recurrent CRLM patients, which has allowed the description of risk factors for survival after a second round of local treatment. Previous studies have provided evidence that repeat hepatic resection is feasible in selected patients with intrahepatic recurrence [4, 5]. However, which individuals would yield maximum benefit from aggressive treatment following first recurrence is not clear, as there is a paucity of data on the risk factors for survival in this patient population. There has been no consensus introduced on how to select candidates.

The present study aimed to investigate prognostic factors for survival and select candidates for the local treatment of CRLM after first recurrence.

## Methods

### Study population

From January 2000 to September 2019, a total of 1027 patients underwent curative hepatic resection for CRLM at the Hepatopancreatobiliary Surgery Department I of Peking University Cancer Hospital. The demographic and clinical data were retrospectively obtained from a prospectively collected patient database. Patients were enrolled in the study based on the following criteria: (1) first recurrence developed after initial radical resection of both the liver metastasis and primary tumor; (2) the initial recurrence was considered resectable and liver-limited by a multidisciplinary team (MDT); (3) no extrahepatic metastasis had occurred since the first hepatic resection; and (4) there were no other simultaneous malignancies. The technical criteria of resectability related to the liver remnant after resection were as follows: (a) anticipated ability to preserve two contiguous segments; (b) anticipated ability to preserve adequate vascular inflow, outflow and biliary drainage; and (c) anticipated ability to preserve an adequate future liver remnant volume (30% in normal livers and 40% in livers pretreated with chemotherapy) [6]. The present study was approved by an Institutional Review Board.

### Preoperative management for first recurrence

At first recurrence, all patients underwent routine laboratory tests, including serum carcinoembryonic antigen (CEA) and carbohydrate antigen (CA) 19–9 levels and liver function tests. Routine imaging modalities, including enhanced computed tomography (CT) scans of the abdominal, thoracic and pelvic regions and hepatic magnetic resonance imaging (MRI), were used to determine the disease stage. Strict criteria were used to detect evidence of cancer recurrence (local, metastatic or secondary colorectal cancer). Recurrence was diagnosed by CT scans, MRI, PET‐CT, or pathology (biopsy or specimen). All imaging reports that suggested recurrence were reviewed carefully and compared to baseline imaging reports to confirm that disease was not present at the time of liver resection. Preoperative chemotherapy was recommended for patients who presented a heavy tumor burden (CRS > 2) and/or recurred within a short period (disease-free survival time of less than 6 months) after the first hepatic resection. The response to chemotherapy was evaluated by MRI according to the Response Evaluation Criteria in Solid Tumors (RECIST 1.1) [7].

### Indications for local treatment

The treatment strategy at first recurrence after hepatic resection was basically the same as that at first hepatic resection; that is, recurrent disease was treated locally only when the overall strategy was considered curative, and all detectable lesions had a tumor-free margin. The resectability was discussed, and clinical treatment decisions were made by an MDT. Systemic chemotherapy was recommended if the patient refused secondary local treatment. Resection of three or more segments was considered major hepatic resection. Percutaneous radiofrequency ablation (RFA) was performed using a CELON system (Teltow, Germany). The bipolar electrode needles were 16G, and guidance ultrasonography was performed using Aloka α-10 (Tokyo, Japan) and GE Logiq E9 (Connecticut, USA) devices. In general, RFA was recommended for deeply located tumors that would require extended resection of the normal parenchyma. RFA was contraindicated when (1) the diameter of the largest tumor exceeded 3 cm and (2) the tumor was adjacent to major bile ducts or large blood vessels or the colon/gallbladder were strictly restricted.

### Postoperative work-up

After completing treatment, all patients underwent regular follow-up examinations with hepatic MRI and CT scans of the abdominal/thoracic/pelvic region, and levels of tumor markers were measured every three months. Adjuvant chemotherapy was usually recommended [8].

### Statistical analysis

Continuous variables are summarized as the means, and categorical variables are summarized as frequencies and percentages. The means of variables were compared with chi square analysis or Fischer’s exact test (depending on the sample size) or with the independent Student’s t test or Mann–Whitney U test, as appropriate. The optimal cutoff points for the definition of early recurrence were determined using the minimum p value approach, which was calculated using the log-rank test for OS after first recurrence. Survival analyses were carried out using the Kaplan–Meier method with the log-rank test. OS was calculated from the date of local treatment or the date of systemic chemotherapy after first recurrence until death or the last follow-up. OS was the primary endpoint for studies with repeat hepatic resection [3, 9]. Cutoff values for continuous variables were determined based on the C-statistic, with a Cox regression model for survival data including censored patients. The estimated cutoff values for each variable were tumor number, 1–5; tumor size, 20–550 mm; CEA level, 5 (upper limit of the normal range), 10, 20, 50, 100, 200 ng/ml; and CA19-9, 37 (upper limit of the normal range), 50, 100 units/ml^7^. All statistical analyses were performed using SPSS 26.0 and R version 3.2.6 (http://www.r-project.org), and p values < 0.05 were considered statistically significant.

### Establishment of nomogram

Univariable and multivariable analyses of various clinicopathological factors by Cox’s proportional hazard model were used to identify independent risk factors for OS in 300 patients. The results of multivariable analysis were used to develop an OS prediction nomogram with 1-, 3-, and 5-year OS as the endpoints. The C-index was calculated to assess the degree of discrimination, and calibration plots were generated to visualize the agreement between the predicted and actual 1-, 3- and 5-year OS with bootstrapped samples. Using all categorical risk factors, we found the best separation in terms of survival by permutation and created two risk groups, a high‐risk and a low‐risk group.

## Results

### Patient characteristics

A total of 867 patients were eligible for study enrollment (Additional file 1: Figure S1). Recurrence was observed in 549 patients (64.1%). Among them, 523 patients (95.3%) developed single-site recurrence [liver (n = 384), lung (n = 86), lymph node (n = 39), and other organ (n = 14)], while 26 patients developed recurrence in multiple organs. Among the patients with single-site recurrence of the liver, 300 were considered to be resectable by an MDT. Repeat hepatic resection and percutaneous radiofrequency ablation were performed in 88 and 85 patients, respectively. The incidence of 90-day mortality was 0% in the repeat resection group. Eight patients underwent intraoperative RFA, and 11 patients underwent R1 resection. There were 66 and 62 patients who received adjuvant chemotherapy in the repeat resection and RFA groups, respectively. The other 127 patients refused local treatment and received only systemic chemotherapy (Table 1). In patients who experienced recurrence, the optimal cutoff point for defining early recurrence was 6 months after hepatic resection based on the results of the minimum p value approach for survival after first recurrence (p = 7.57 × 10^–17^) (Additional file 2: Figure S2).

**Table 1:** Demographic and clinical characteristics of patients after first recurrence

Variable	Local treatment		Systemic chemotherapy	P value
	Resection	RFA		
Patients demographics	n = 88	n = 85	n = 127	
Age (years)	55.6 ± 10.3	57.7 ± 9.3	56.1 ± 9.8	0.75
Sex ration (M:F)	57:31	59:26	80:47	0.83
Primary T				
T1-2	7 (8.0%)	7 (8.2%)	18 (14.2%)	0.23
T3-4	81 (92.0%)	78 (91.8%)	109 (85.8%)	
Primary N				
N0	54 (61.4%)	66 (77.6%)	94 (74.0%)	0.82
N1-2	34 (38.6%)	19 (22.4%)	33 (26.0%)	
Primary tumor location				
Colon	55 (62.5%)	52 (61.2%)	78 (31.4%)	0.95
Rectum	33 (37.5%)	33 (38.8%)	49 (38.6%)	
Right side	24 (27.3%)	17 (20.0%)	34 (26.8%)	0.75
Left side	64 (72.7%)	68 (80.0%)	93 (73.2%)	
Timing of liver metastasis				
Synchronous	72 (81.8%)	78 (91.8%)	110 (86.6%)	0.53
Metachronous	16 (18.2%)	7 (8.2%)	17 (13.4%)	
Metastasis no	1 (1–7)	1 (1–3)	2 (1–5)	0.00
Metastasis size(mm)	23.1 ± 15.8	15.6 ± 1.7	19.6 ± 10.2	0.00
Localization of liver metastases				
Unilobar	63 (71.6%)	70 (82.4%)	72 (56.7%)	0.00
Bilobar	25 (28.4%)	15 (17.6%)	55 (43.3%)	
CEA > 50	13 (14.8%)	4 (4.7%)	5 (3.9%)	0.00
CA199 > 100	10 (11.4%)	6 (7.1%)	8 (6.3%)	0.18
Ras status				
Wild	62 (70.5%)	50 (58.8%)	77 (60.6%)	0.49
Mutation	26 (29.5%)	35 (41.2%)	50 (39.4%)	
Preoperative chemotherapy	49 (55.7%)	25 (29.4%)	127 (100%)	NA
Up-front local treatment	39 (44.3%)	60 (70.6%)	0	
No. of lines				
First line	25 (28.4%)	12 (14.1%)	70 (55.1%)	NA
Second line	24 (27.3%)	13 (15.3%)	47 (37.0%)	
Others	0	0	10 (7.9%)	
Regimen				
Oxaliplatin	18 (20.5%)	9 (10.6%)	49 (38.6%)	NA
Irinotecan	31 (35.2%)	16 (18.8%)	70 (55.1%)	
Others	0	0	8 (6.3%)	
Biological agents	39 (44.3%)	20 (23.5%)	95 (74.8%)	NA
Bevacizumab	29 (33.0%)	18 (21.2%)	70 (55.1%)	
Cetuximab	10 (11.3%)	2 (2.3%)	25 (19.7%)	
Response to chemotherapy				
Complete	0	0	0	NA
Partial	4 (4.5%)	2 (2.4%)	21 (16.5%)	
Stable disease	45 (51.1%)	23 (27.1%)	89 (70.1%)	
Progressive disease	0	0	17 (13.4%)	
Intraoperative RFA	8 (9.1%)	NA	NA	
Operation time	204.4 ± 89.0	NA	NA	NA
Blood lose	274.1 ± 77.1	NA	NA	NA
RBC transfusion	2	NA	NA	NA
R1 resection	6	NA	NA	NA
Complication	4	NA	NA	NA
Major	1	0	NA	NA
Adjuvant chemotherapy	66 (75%)	62 (73.0%)	NA	NA

### Survival analysis

**Fig. 1:**
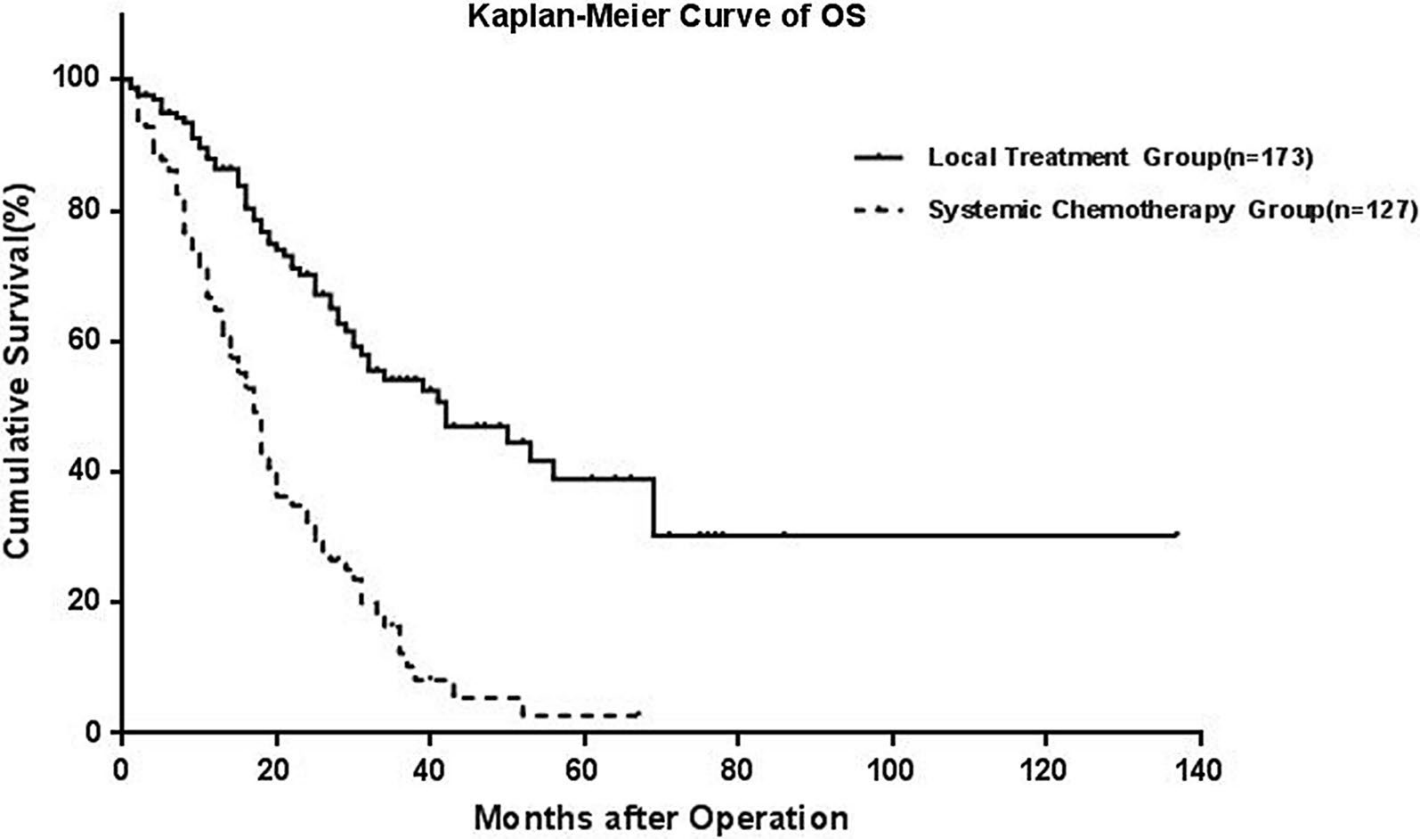
The OS of local treatment and chemotherapy was shown by a Kaplan-Meier curve

The median follow-up was 39 months after first recurrence (95% CI: 35–43 months). The 1-, 3- and 5-year OS and DFS rates were 92.3%, 59.3%, and 46.2% and 46.8%, 28.4%, and 25.4%, respectively, for 867 CRLM patients (Additional file 3: Figure S3). The 1-, 3- and 5-year OS rates for the local treatment group were 86.3%, 54.1% and 38.9%, respectively, and those for the systemic chemotherapy group were 64.8%, 16.3% and 2.7%, respectively. The difference between the two groups was significant (p < 0.001) (Fig. 1). Of the 173 patients who underwent local treatment, the 5-year OS and DFS rates were 38.9% and 22.2%, respectively (Additional file 4: Figure S4). Among them, 97 patients (56.1%) developed recurrence after local treatment.

### Prognostic factors for OS after first recurrence

In univariable analysis, primary N + , tumor number > 1, largest tumor size > 3 cm, RAS mutation, early recurrence, bilateral distribution at first recurrence and no local treatment were related to decreased OS (p < 0.05). Five independent prognostic factors for OS were identified in multivariable analysis: node-positive primary (HR = 1.857, 95% CI: 1.227–2.809; p = 0.003), tumor size > 3 cm (HR = 1.707, 95% CI: 1.037–2.810; p = 0.036), early recurrence (HR = 1.693, 95% CI: 1.142–2.511; p = 0.009), RAS mutation (HR = 1.553, 95% CI: 1.080–2.234; p = 0.017) and local treatment (HR = 0.322, 95% CI: 0.222–0.467; p < 0.001) (Table 2).

**Table 2:** Univariable and multivariable analysis of factors associated with OS of CRLM after first recurrence

	Univariable analysis			Multivariable analysis		
	HR	95%	P value	HR	95%	P value
Age						
> 70	Ref	0.869–2.461	0.153	Ref	0.777–2.366	0.284
≤ 70	1.462			1.356		
Sex						
Male	Ref	0.670–1.322	0.726	Ref	0.586–1.201	0.336
Female	0.941			0.838		
Primary T stage						
1–2	Ref	0.515–1.355	0.465	Ref	0.502–1.424	0.529
3–4	0.835			0.846		
Primary N stage						
N0	Ref	1.265–2.734	0.002	Ref	1.227–2.809	0.003
N1-2	1.860			1.857		
Location tumor						
Colon	Ref	0.600–1.159	0.279	Ref	0.517–1.187	0.249
Rectum	0.834			0.783		
Primary tumor location						
Left	Ref	0.834–1.734	0.322	Ref	0.770–1.973	0.383
Right	1.203			1.233		
Timing of liver metastasis						
> 12 month	Ref	0.588–1.435	0.708	Ref	0.613–1.644	0.970
≤ 12 month	0.981			1.010		
CEA						
> 50	Ref	0.451–1.443	0.455	Ref	0.408–1.515	0.472
≤ 50	0.798			0.786		
CA199						
> 100	Ref	0.675–1.974	0.600	Ref	0.690–2.333	0.444
≤ 100	1.154			1.269		
Tumor size						
≤ 3 cm	Ref	1.074–2.222	0.035	Ref	1.037–2.810	0.036
> 3 cm	1.441			1.707		
Tumor no						
≤ 1	Ref	1.006–1.942	0.046	Ref	0.424–1.268	0.733
> 1	1.398			0.733		
RAS status						
Wild	Ref	1.183–2.281	0.003	Ref	1.080–2.234	0.017
Mutation	1.634			1.553		
Distribution						
Unilobar	Ref	1.094–2.205	0.014	Ref	0.806–2.380	0.238
Bilobar	1.553			1.385		
Early recurrence						
No	Ref	1.387–2.725	0.000	Ref	1.148–2.523	0.008
Yes	1.944			1.702		
Local treatment						
No	Ref	0.222–0.443	0.000	Ref	0.218–0.467	0.000
Yes	0.313			0.322		

### Prognostic nomogram and calibration for OS

**Fig. 2:**
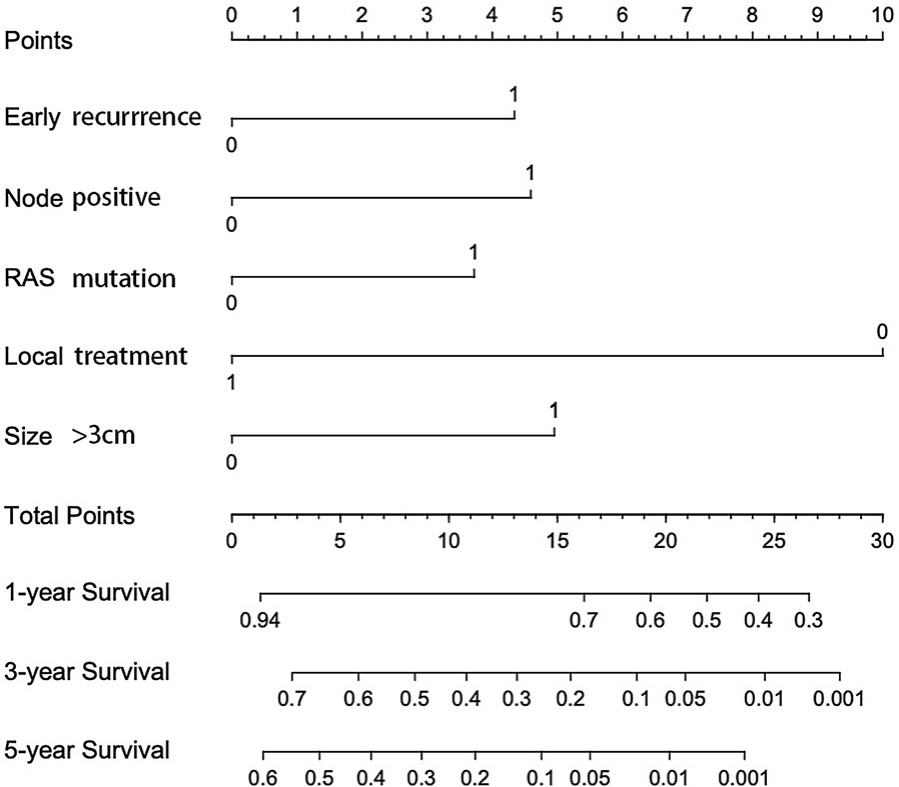
Colorectal liver metastasis nomogram for OS after first recurrence

**Fig. 3:**
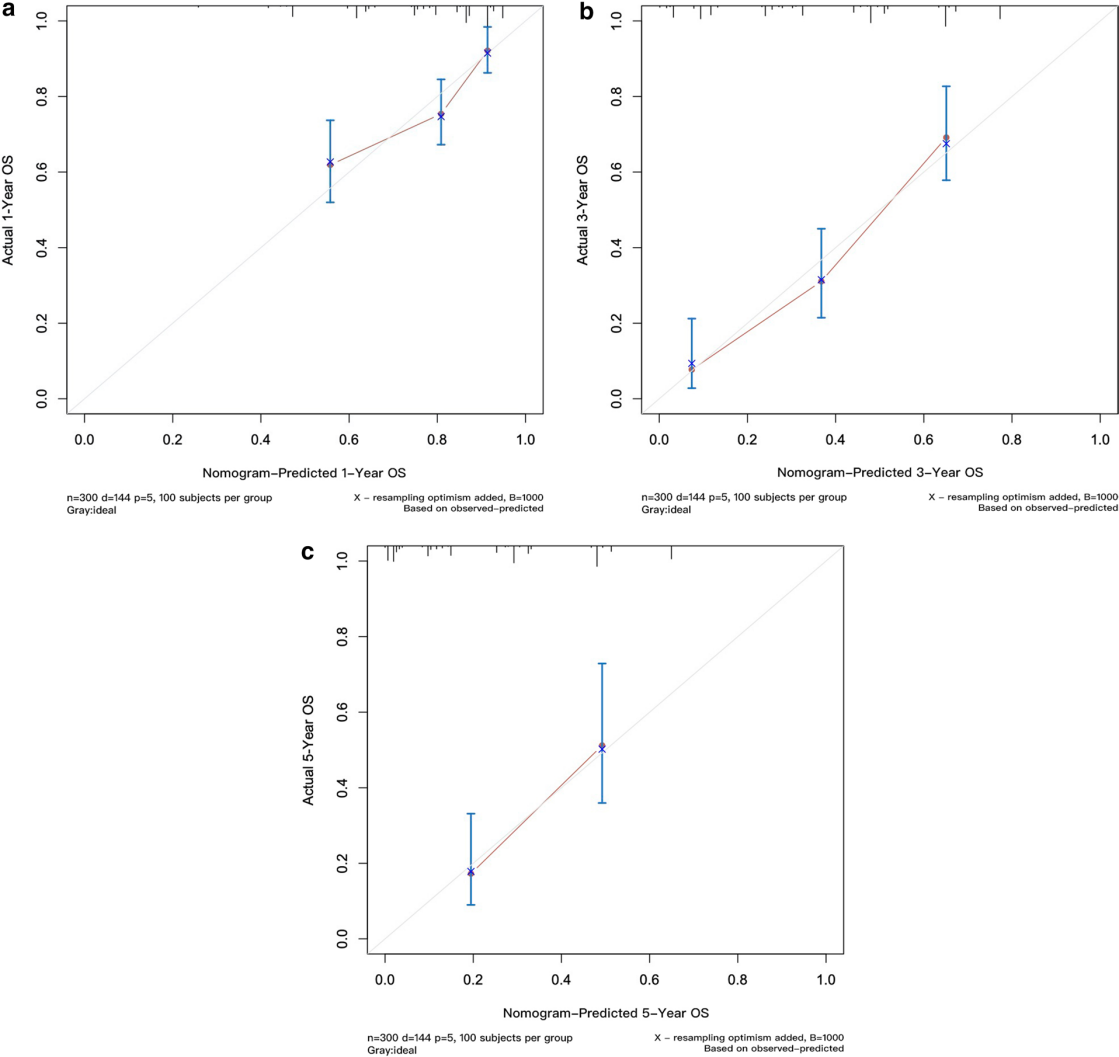
**a** The predicted OS at 1 year by a calibration curve. **b** The predicted OS at 3 years by a calibration curve. **c** The predicted OS at 5 years by a calibration curve

We developed a point-based prognostic nomogram to predict OS at first recurrence based on the five independent prognostic factors (Fig. 2). The sum of each score is presented in Additional file 5: Table S1. The C-index of the prognostic nomogram was 0.707 for predicting OS. The calibration plot suggested that the accuracy of the predicted 1-, 3-, and 5-year OS was excellent (Fig. 3a–c).

### Performance of the nomogram for predicting OS

In the development cohort, the AUCs of the nomogram score for predicting OS at 1, 3, and 5 years were higher than those of the algorithms recommended by Hof [10], Neal [11], and Serrano[12] (Additional file 6: Figure S5 and Additional file 7: Table S2).

### Stratification by nomogram and indications for repeat local treatment

The survival curves stratified by quartiles of the nomogram-predicted score are shown in Additional file 8: Figure S6. Compared with patients (total score ≥ 13) who received systemic chemotherapy, radical local treatment did not significantly improve survival outcomes compared to those before treatment (median OS: 21 vs. 15 months, p = 0.126) (Additional file 9: Figure S7).

## Discussion

Curative resection for CRLM improves survival outcomes and curative opportunities; however, the majority of patients will develop recurrence [13]. It has been suggested that secondary hepatic resection is a safe and feasible procedure for recurrent CRLM patients. However, repeat hepatic resection may not be possible due to anatomical or functional restraints [9, 10]. RFA is a useful alternative in this situation. The indications for repeat hepatic resection were believed to be the same as those for initial resection [4, 14]. However, the disease characteristics were not exactly compared between the initial and recurrent resection time points.

The indication and concerning prognostic factors for the local treatment of first recurrence have not been well studied or defined. Previous analyses have been limited by small and single cohorts of patients [4, 14]. In the present study, 173 patients who underwent local treatment at first recurrence for CRLM and had significantly better survival outcomes than those who only received systemic chemotherapy. Rumor size > 3 cm, node-positive primary, early recurrence, and RAS mutation have been identified to be risk factors for survival outcomes CRLM patients at first recurrence. Furthermore, in the high-risk group (total score ≥ 13), local treatment did not significantly improve OS (median OS: 21 vs. 15 months, p = 0.126). The present model might facilitate the identification of patients who may benefit most from repeat local treatment before an individualized treatment decision is made.

Recurrence occurs in 60–84% of CRLM patients after initial hepatic resection [15, 16]. The recent expansion of indications for surgery has led to an increase in the number of patients with potentially resectable disease, while these factors can also lead to an increased risk of early recurrence. Early recurrence has been reported to adversely influence survival after hepatic resection and therefore remains a concern for repeat hepatic resection because of worse patient prognosis [17, 18]. The present study investigated whether the optimal cutoff point for early recurrence was 6 months. Although it remained an independent prognostic risk factor, aggressive treatment should therefore be proactively considered even for patients with early recurrence. If the patient was determined to have unresectable early recurrence, systemic chemotherapy should be recommended. For patients with resectable disease, radical resection should be performed as often as possible. Two cycles of neoadjuvant chemotherapy should be recommended, followed by local treatment. If there is a risk of removing liver metastases, the patient should undergo upfront resection directly. RFA should be performed if the tumor is located deep in the liver.

The liver and lungs are the predominant sites of secondary recurrence, of which liver metastases are especially common [3, 19]. Therefore, the present study enrolled only patients with resectable, liver-limited disease at first recurrence. Repeat hepatic resection for recurrence has been reported to be associated with an equivalent long-term outcome to first hepatic resection, with a similarly low surgical risk, and the 5-year OS rate ranged from 27 to 45% [20–23]. RFA has emerged as an alternative radical treatment with lower invasiveness and a lower complication rate and is effective for patients with comorbidities and recurrent liver disease [24, 25]. Previous studies of percutaneous RFA for recurrent CRLM yield similar results [26, 27]. It has been suggested that tumor diameter and number are the most important factors that influence the efficacy [28, 29]. In our study, RFA was recommended in deeply located tumors that would require extensive resection of the normal parenchyma.

Given that the survival of patients who undergo liver resection for colorectal cancer metastases is long, surgeons and medical oncologists are now dealing with a “chronic disease” that should be treated differently depending on its presentation upon recurrence [30]. In the modern era, in addition to the current disease state of CRLM patients, recurrence should be considered when determining the treatment strategy. Preoperative chemotherapy might shrink tumors and increase resectability. In particular, it also likely selected individuals with recurrence who would benefit from resection. Therefore, this treatment might be recommended for patients who develop early recurrence or have a heavy disease burden in the liver.

### Limitations

The limitations of the present study included its retrospective design and the gradual change in indications for resection and RFA over the course of the study. First, although the enrolled patients were determined to have resectable disease, bias could still have existed from patient selection in the three groups. Therefore, these results should be validated with further better quality studies. Second, it is possible that other unknown factors could affect the accuracy of the nomogram model and that a higher number of cases might be required to be detect them. This type of study is difficult to perform prospectively, and any trials must be very carefully performed. Moreover, there was no external validation cohort in the present study. Finally, all data were collected from a limited number of institutions.

## Conclusion

Radical treatment remains the gold-standard for recurrent colorectal liver metastasis. The proposed model may help to predict the possibility of radical interventions (score < 13) and provide optimal individualized treatment.

## Supplementary Information

**Additional file 1: Figure S1.** Flowchart of the enrolled cohorts.

**Additional file 2: Figure S2.** The definition of early recurrence using the minimum p value approach by an optimal cutoff point regarding the time to first recurrence.

**Additional file 3: Figure S3.** Kaplan-Meier curve showing the OS and DFS of 867 patients.

**Additional file 4: Figure S4.** Kaplan-Meier curve showing the OS and DFS of local treatment.

**Additional file 5: Table S1.** Prognostic factors.

**Additional file 6: Figure S5.** The OS of the high-risk (score ≥ 13) and low-risk groups (score < 13) by a Kaplan-Meier curve.

**Additional file 7: Table S2.** Comparison of different models for predicting OS.

**Additional file 8: Figure S6.** The OS of local treatment and chemotherapy in the high-risk group by a Kaplan-Meier curve.

**Additional file 9: Figure S7.** Kaplan-Meier curve showing the OS of local treatment and chemotherapy in the high-risk group.

## Data Availability

The datasets generated and/or analyzed during the current study are not publicly available to protect individual patient privacy but are available from the corresponding author upon reasonable request.

## Abbreviations

CRLM: Colorectal liver metastasis
RFA: Radiofrequency ablation
OS: Overall survival
DFS: Disease-free survival
MDT: Multidisciplinary team
CEA: Carcinoembryonic antigen
HR: Hazard ratio
MRI: Magnetic resonance imaging
CT: Computed tomography
