# Supplementary figures and images for: Recurrent colorectal liver metastasis patients could benefit from repeat hepatic resection
Source: BMC Surg. 2021 Aug 16;21:327. doi: 10.1186/s12893-021-01323-y (PMC8365902; doi:10.1186/s12893-021-01323-y)

Supplementary Figure 1. Flow Chart of Enrolled Patients.

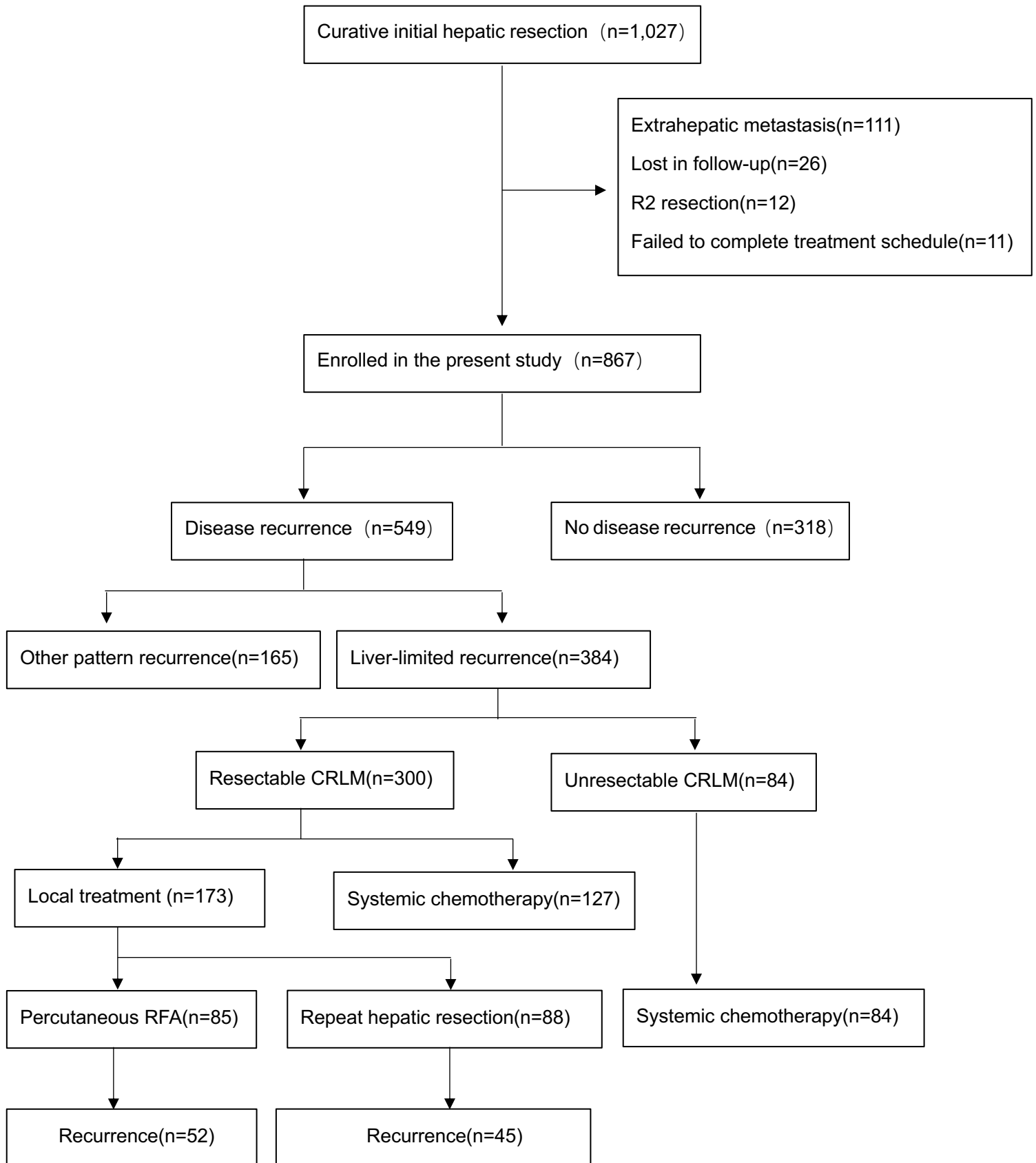

Supplement: Supplementary file 1 — Additional file 1: Figure S1. Flowchart of the enrolled cohorts. [file 12893_2021_1323_MOESM1_ESM.pdf]

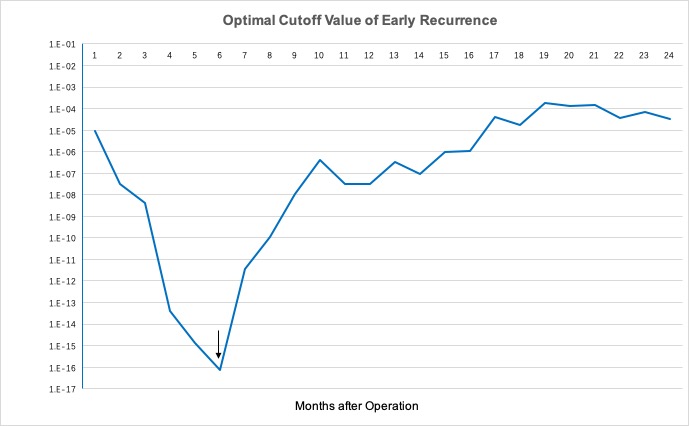

Supplement: Supplementary file 2 — Additional file 2: Figure S2. The definition of early recurrence using the minimum p value approach by an optimal cutoff point regarding the time to first recurrence. [file 12893_2021_1323_MOESM2_ESM.docx]

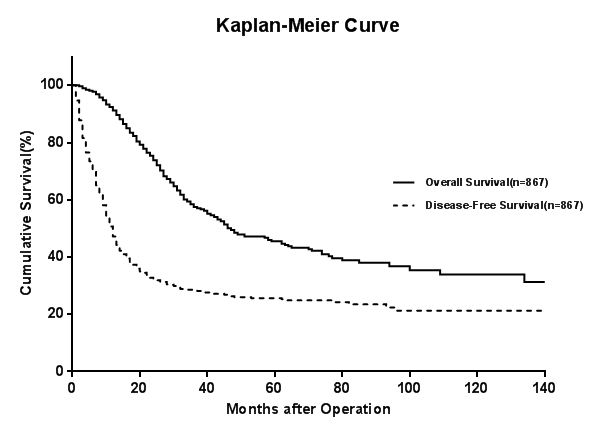

Supplement: Supplementary file 3 — Additional file 3: Figure S3. Kaplan-Meier curve showing the OS and DFS of 867 patients. [file 12893_2021_1323_MOESM3_ESM.docx]

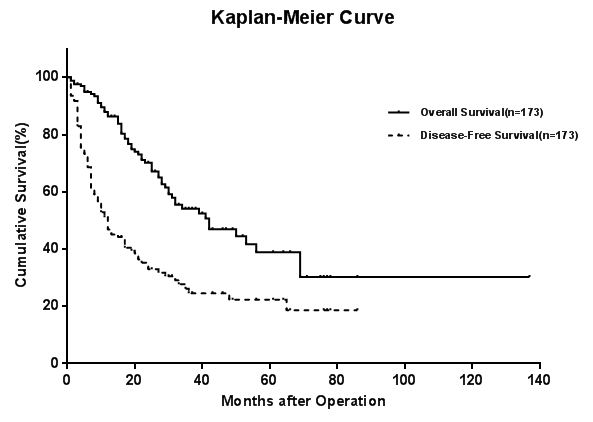

Supplement: Supplementary file 4 — Additional file 4: Figure S4. Kaplan-Meier curve showing the OS and DFS of local treatment. [file 12893_2021_1323_MOESM4_ESM.docx]

**
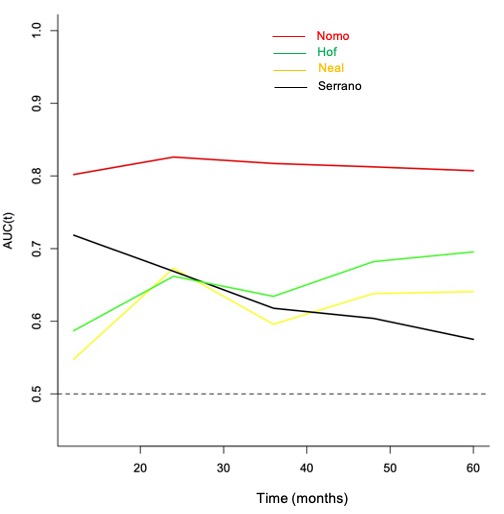
**

Supplement: Supplementary file 6 — Additional file 6: Figure S5. The OS of the high-risk (score ≥ 13) and low-risk groups (score < 13) by a Kaplan-Meier curve. [file 12893_2021_1323_MOESM6_ESM.docx]

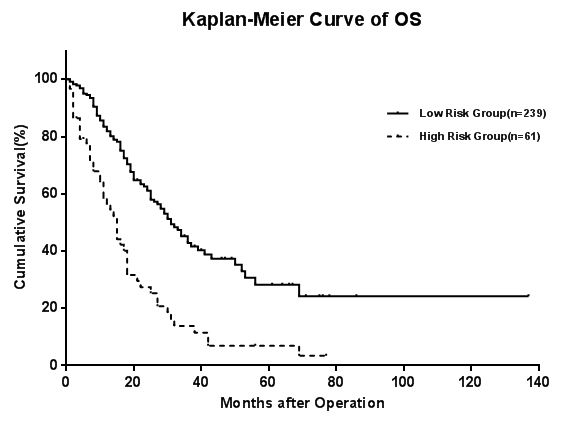

Supplement: Supplementary file 8 — Additional file 8: Figure S6. The OS of local treatment and chemotherapy in the high-risk group by a Kaplan-Meier curve. [file 12893_2021_1323_MOESM8_ESM.docx]

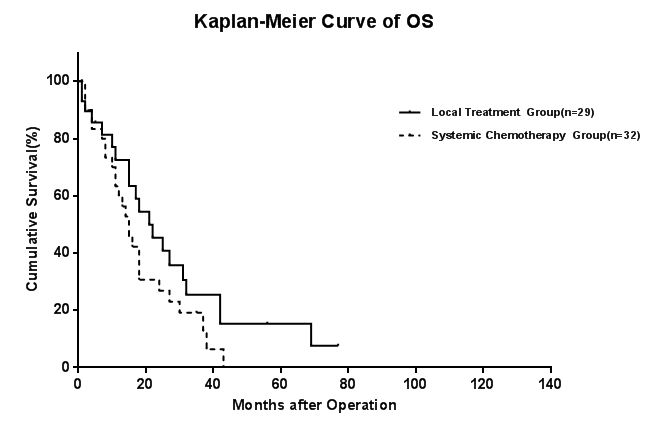

Supplement: Supplementary file 9 — Additional file 9: Figure S7. Kaplan-Meier curve showing the OS of local treatment and chemotherapy in the high-risk group. [file 12893_2021_1323_MOESM9_ESM.docx]
