# Supplementary material for: Recurrent colorectal liver metastasis patients could benefit from repeat hepatic resection
Source: BMC Surg. 2021 Aug 16;21:327. doi: 10.1186/s12893-021-01323-y (PMC8365902; doi:10.1186/s12893-021-01323-y)
Supplement: Supplementary file 5 — Additional file 5: Table S1. Prognostic factors. [file 12893_2021_1323_MOESM5_ESM.docx]

**Supplementary Table 1. Specific score of risk factors**

| **Factor** | **Score** |
| --- | --- |
| Primary tumor N category |  |
| N0 | 0 |
| N+ | 5 |
| Tumor size hepatic resection(cm) |  |
| ≤3 | 0 |
| >3 | 5 |
| RAS status |  |
| Wild type | 0 |
| Mutation type | 4 |
| DFS after firs hepatic resection(m) |  |
| ≤6 | 4 |
| >6 | 0 |
| Radical local treatment |  |
| Yes | 0 |
| No | 10 |
