# Supplementary material for: Recurrent colorectal liver metastasis patients could benefit from repeat hepatic resection
Source: BMC Surg. 2021 Aug 16;21:327. doi: 10.1186/s12893-021-01323-y (PMC8365902; doi:10.1186/s12893-021-01323-y)
Supplement: Supplementary file 7 — Additional file 7: Table S2. Comparison of different models for predicting OS. [file 12893_2021_1323_MOESM7_ESM.docx]

**Supplemental Table 2. Comparison of different models in predicting OS**

| Models in cohorts | AUC (95% CI) | | |
| --- | --- | --- | --- |
|  | 1-year OS | 3-year OS | 5-year OS |
| Primary cohort |  |  |  |
| The current nomogram | 0.802 (0.719-0.885) | 0.817 (0.734-0.876) | 0.807 (0.724-0.891) |
| Hof | 0.587 (0.504-0.702) | 0.634 (0.551-0.709) | 0.695 (0.612-0.796) |
| Neal | 0.548 (0.464-0.642) | 0.596 (0.512-0.671) | 0.641 (0.557-0.753) |
| Serrano | 0.718 (0.635-0.813) | 0.618 (0.535-0.692) | 0.575 (0.492-0.686) |

AUC indicates area under the (time-dependent) receiver operating characteristic curve.
